# Balancing *Varroa* management and honey bee resilience: Behavioral and physiological consequences of temporarily high mite pressure

**DOI:** 10.1016/j.ijppaw.2025.101137

**Published:** 2025-09-13

**Authors:** Lioba Hilsmann, Markus Krischke, Martin J. Mueller, Sarah Manzer, Ricarda Scheiner

**Affiliations:** a Behavioral Physiology and Sociobiology, Biocenter, Julius-Maximilians-Universität Würzburg, Am Hubland, 97074, Würzburg, Germany; b Pharmaceutical Biology, Biocenter, Julius-von-Sachs-Institute, Julius-Maximilians-Universität Würzburg, Julius-von-Sachs-Platz 2, 97082, Würzburg, Germany

## Abstract

Pollinators are essential for global agriculture and ecosystem stability, yet many populations are declining due to parasites and pathogens. Among these, the ectoparasitic mite *Varroa destructor* is one of the most critical challenges to honey bees (*Apis mellifera*). Conventional treatment approaches use frequent interventions to keep mite levels as low as possible, whereas other approaches aim to promote natural selection by omitting treatments. A possible compromise lies in reducing treatments while maintaining colony survival through targeted interventions. This approach may allow adaptive responses under temporary mite pressure. In this study, we compared two beekeeping strategies: 1) Conventional beekeeping practice involving regular drone brood removal during mating season, formic acid treatment in summer, and oxalic acid treatment in winter. 2) An innovative approach where drone brood is left in the colony and a summer brood interruption is induced, followed by an oxalic acid treatment. Winter treatment is only applied if *Varroa* pressure exceeds three naturally dropped mites per day shortly before winter treatment. We investigated *Varroa* infestation and its consequences for honey bee foraging behavior, homing ability, juvenile hormone III levels, pollen protein content, and honey yield. Bees from innovatively managed colonies started foraging earlier and had elevated juvenile hormone levels at young ages. Orientation ability was unaffected, but these bees performed longer foraging trips and collected pollen with higher protein content. They also stopped foraging earlier, likely reflecting a reduced lifespan due to increased *Varroa* pressure. Nevertheless, colony productivity did not differ between the two beekeeping approaches. Our findings suggest that reduced *Varroa* treatments and temporarily high mite pressure do not have negative effects on colony performance. Such approaches may offer a potential middle ground between intensive conventional management and selection-based strategies, balancing colony vitality and the possibility of fostering resistance traits through controlled exposure of parasite and host.

## Highlights

• Reduced *Varroa* treatments allow adaptive traits without harming colony performance.
• Bees under temporary mite pressure forage earlier with elevated juvenile hormone levels.
• Innovatively managed bees perform longer trips, but homing ability remains unaffected.
• Mite-exposed foragers collect higher-protein pollen.

## Graphical abstract

**Figure:**
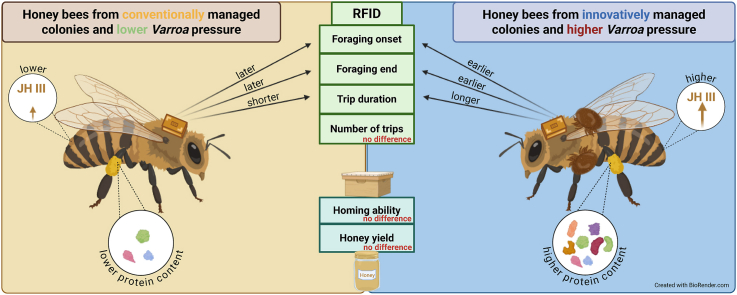

## 1 Introduction

Honey bees are among the most important pollinators worldwide, playing a crucial role for biodiversity maintenance and agricultural productivity (Klein et al., 2007). The ecological and economic significance of honey bees is closely linked to their highly evolved eusocial life, which is characterized by a sophisticated division of labor, cooperative brood care, and a distinct reproductive hierarchy between queen and workers (Crespi and Yanega, 1995; Queller and Strassmann, 2003). This complex social organization makes honey bees ideal model organisms for studying the close interplay between individual and collective processes within a colony. It also provides insights into mechanisms of self-organization and colony-level compensation in response to changing environmental conditions and external stressors (Ulgezen et al., 2021).

Since the host shift of the ectoparasitic mite *Varroa destructor* from Eastern honey bees (*Apis cerana*) to Western honey bees (*Apis mellifera*), colonies have been exposed to a novel stressor to which they are poorly adapted (Rosenkranz et al., 2010; Traynor et al., 2020). Unlike its original host, *A. mellifera* lacks effective behavioral and physiological defense mechanisms, such as *Varroa*-sensitive hygiene and efficient recapping behavior (Grindrod and Martin, 2023). Beyond direct damage caused by mite feeding, *Varroa* acts as a vector for honey bee viruses, such as Deformed Wing Virus and Acute Bee Paralysis Virus, which drastically accelerate colony decline (Traynor et al., 2020; Hilsmann et al., 2025). This disease complex, often referred to as varroosis, makes infestations a major driver for colony losses worldwide (Oldroyd, 1999; Grindrod and Martin, 2023).

*Varroa* profoundly affects both the behavior and physiology of infested honey bees. Mites weaken their hosts directly by feeding on hemolymph and fat body tissue, resulting in impaired immune function and increased susceptibility to pathogens (Yang and Cox-Foster, 2007; Ramsey et al., 2019; Traynor et al., 2020; Han et al., 2024). Physiological consequences include reduced body weight, reduced lifespan, and diminished flight performance (De Jong et al., 1982; Schneider and Drescher, 1987; Amdam et al., 2004). On a behavioral level, parasitized workers show deficits in non-associative learning and altered foraging activity (Kralj and Fuchs, 2006; Kralj et al., 2007). Additionally, *Varroa* infestation has been associated with an accelerated transition from nursing to foraging tasks, which correlates with precocious behavioral maturation and premature senescence, ultimately reducing colony productivity (Janmaat and Winston, 2000; Nazzi et al., 2012; Zanni et al., 2018). These combined effects significantly contribute to colony weakening and increases the risk of colony loss.

To control *Varroa* infestations and to reduce their impact on honey bee colonies, beekeepers commonly apply a range of treatments against *Varroa* throughout the year. These include drone brood removal during mating season, because of the mite's preference for drone brood due to the longer development time and stronger olfactory cues from drone larvae and their larval food (Fuchs, 1990; Le Conte et al., 1989; Nazzi et al., 2004). Additionally, treatments with organic acids such as formic acid in summer and oxalic acid during the broodless period in winter are used to reduce mite infestation throughout the year (Brodschneider et al., 2022). These interventions can lower *Varroa* populations to a level that reduces colony damage when applied effectively (Bachert and Scheiner, 2023). However, consistent suppression of parasite pressure on the host may also limit potential for natural selection and development of adaptive traits in honey bee populations, such as increased grooming behavior or hygienic brood removal, which could enhance the long-term resilience of honey bee populations to *Varroa* (Rinkevich, 2020). Complete absence of mite control, thereby exposing colonies to natural selection pressure, would lead to losses of most honey bee colonies within two years (Rosenkranz et al., 2010; Neumann and Blacquière, 2017). Therefore, we investigated a method that aimed to strike a balance between the two extremes and compared it to the conventional approach. Our “innovative” approach combined induced brood interruption and reduced *Varroa* treatments. (1) In spring, we omitted drone brood removal, thereby allowing *Varroa* to reproduce and increase parasite pressure on the host. (2) Classical summer treatment with formic acid was replaced by an induced brood interruption followed by a single oxalic acid treatment. (3) Winter treatment with oxalic acid was only applied when infestation thresholds exceeded three naturally dropped mites a day, measured shortly before the planned winter treatment.

This beekeeping practice resulted in controlled *Varroa* pressure during spring and summer, achieved by omitting drone brood removal and summer treatment with induced brood interruption followed by a single oxalic acid treatment in summer. These interventions may support the development of resistance traits in honey bee populations in the long run, while ensuring sufficient production of resilient and healthy winter bees.

In this study, we investigated whether temporarily increased *Varroa* pressure in spring and summer affects key physiological and behavioral traits of honey bees. Specifically, we examined whether elevated mite infestation levels influenced foraging and flight activity, using radio frequency identification based monitoring of individual bees from both beekeeping approaches to investigate the flight and foraging behavior. Additionally, we tested whether temporarily high *Varroa* pressure during active season affects the orientation and homing abilities of bees. On a physiological level, we examined changes in juvenile hormone III titers, because this hormone plays a key role in regulating behavioral maturation and longevity. Furthermore, we analyzed the nutritional quality of pollen collected by foragers, which is essential for successful rearing of healthy honey bees. Our aim was to evaluate potential trade-offs between fostering natural resistance traits in the long run and maintaining colony performance and vitality under reduced *Varroa* treatments.

## 2 Materials and methods

### 2.1 Honey bees

Honey bee colonies (*Apis mellifera carnica*) were located at the departmental apiary of the Universität Würzburg in Southern Germany. All colonies were established with open-mated sister queens, thereby reducing potential genetic variability between colonies. Five colonies were assigned to each beekeeping scheme. Details of treatment schemes are listed in Table S2 in the supplementary material. To prevent potential queen loss and ensure colony stability, all colonies were requeened with new open-mated sister queens in summer 2022. In innovatively managed colonies, requeening took place after queen-caging, whereas in conventionally managed colonies, new queens were introduced before summer treatment.

In the conventional approach, drone brood was removed in spring and summer. The summer treatment with formic acid (Formivar®, 60 % Andermatt BioVet GmbH, Germany) was carried out in late summer after honey harvest, by using a Nassenheider Professional® evaporator on top of the colonies (Joachim Weiland, Werkzeugbau GmbH & Co KG, Germany). In addition, the winter treatment with oxalic acid (Oxuvar®, 5.7 %, Andermatt BioVet GmbH, Germany) was trickled onto the winter cluster (see Table S2 for exact timing).

In the innovative approach, drones were allowed to develop in the colony, and summer treatment against *Varroa* was performed biotechnically by caging the queen in an API-MO.BRU®-cage (API-MO.BRU, Mozzato Bruno, Italy). This flexible thermoplastic cage prevents the queen from laying eggs while allowing worker bee access. It fits into a standard comb frame, and measures approximately 5 x 7.8 × 3 cm (width x height x depth). Queens were caged for 25 days until colonies were brood-free. Afterwards, oxalic acid (Oxuvar®, 5.7 %, Andermatt BioVet GmbH, Germany) was trickled on adult honey bees at night when all foragers were in colonies to reduce the *Varroa* load (for exact timing of all treatments see Table S2). Oxalic acid winter treatment was unnecessary for innovatively managed colonies, as *Varroa* infestation did not exceed three mites naturally dropped on sticky bottom boards per day shortly before planned winter treatment.

In summary, the main difference between the two methods is that in the innovative beekeeping approach, there were fewer treatments against *Varroa* compared to the conventional beekeeping scheme, naturally leading to a higher mite infestation load in spring and summer to support a possible adaptation of honey bees to their parasite.

### 2.2 Daily mite drop on sticky bottom boards

To monitor *Varroa* infestation of colonies, sticky bottom boards were placed under colonies for two days, at least every two weeks. The boards were covered with a thin layer of rapeseed oil to prevent predation of dead mites by ants and other insects. After two days, the number of dead mites on boards was counted by eye and divided by two to calculate the daily mite drop. This parameter is commonly used as a proxy for the overall *Varroa* population within the colony, as the number of naturally dead mites dropping onto bottom boards correlates with colony infestation levels (Martin, 1998; Medina-Flores et al., 2024). Monitoring of mite drop was measured from July 2021 until July 2024.

### 2.3 Radio frequency identification experiments

From each beekeeping method, two capped brood frames were taken, each originating from a different colony and stored overnight in an incubator (32 °C, 70 % RH). The next day, all newly emerged honey bees were removed from the brood frames and stored in different boxes depending on the beekeeping scheme. Individual bees were immobilized on ice and fixed to styrofoam plates with crossed needles between the thorax and abdomen. Radio frequency identification tags (mic3-TAG 16k, Microsensys GmbH), which were scanned first with a USB pen to assign the IDs, were glued with super glue (UHU® Sekundenkleber blitzschnell Pipette) to the thorax of the honey bees. After drying, honey bees were transferred to cages depending on the beekeeping method and fed with 50 % sucrose solution *ad libitum*. Cages were placed on top of four separate Mini-Plus colonies (280 x 280 × 270 mm, each containing six frames measuring 251 × 159 mm) that were used for experiments. One hundred innovatively and 100 conventionally managed bees were introduced to each Mini-Plus colony. In June 2022, four replicates were conducted, each with 100 tagged honey bees per treatment and colony, followed by two additional replicates in June 2023 under the same conditions. For each replicate, different source colonies were used, depending on which colonies provided capped brood frames with emerging bees at the time. Data were collected until no additional tagged bees were detected.

### 2.4 Juvenile hormone III analysis

For juvenile hormone III analysis, capped brood frames were obtained from colonies managed under both beekeeping methods. For each replicate, two capped brood frames were taken from two different colonies per beekeeping method, depending on which colonies provided capped brood close to emergence at the respective time. Frames were stored overnight in an incubator (32 °C, 70 % RH). The next day, all newly emerged honey bees were brushed carefully with a honey bee broom into separate boxes depending on the beekeeping method. Newly emerged honey bees were color-marked and transferred to cages where they were fed with 50 % sucrose solution *ad libitum*. Cages were placed on top of the four Mini-Plus colonies that were used for the experiments. After 6, 8, 10, and 12 days, 10 honey bees were sampled from each treatment and immobilized on ice. After fixing them with needles onto a styrofoam plate, a glass microcapillary (servoprax®, A1 0115, servoprax GmbH) was used to pierce the cuticle between the fourth and fifth abdominal segment. Up to 5 μl of hemolymph were sampled, flash-frozen immediately in liquid nitrogen, and stored at −80 °C until analysis. Juvenile hormone III levels in the hemolymph were determined using liquid chromatography-tandem mass spectrometry (LC-MS/MS). Analysis was performed with a Waters Acuity ultrahigh-performance liquid chromatography system coupled to a Waters Micromass Quattro Premier triple quadrupole mass spectrometer (Milford, MA), following the methodology described before by Scholl et al. (2014) and Schilcher et al. (2021). This experiment was carried out four times, with two replicates in June 2023 and two replicates in July 2024.

### 2.5 Homing experiments

For homing experiments, 50 foragers were captured directly at the colony entrance as they exited their colonies. For each replicate, one colony from the innovative management and one colony from the conventional management were used, resulting in 50 foragers from each treatment per replicate. After being immobilized on ice, each honey bee was marked with a colored dot on the thorax for individual identification and gently mounted in metal holders. Subsequently, the bees were fed with a 60 % sucrose solution until they were considered satiated. Satiation was determined by repeatedly touching their antennae with the solution and was reached when the bees no longer exhibited a proboscis extension response. A 60 % sucrose solution was used to ensure that the bees returned immediately after release. After feeding, the honey bees were placed in small cages and transported to a release site approximately 500 m away from the apiary. Upon release, the time taken by each bee to return to its colony was recorded. To prevent double-counting, a mesh screen was installed at the colony entrance, allowing returning marked bees to be collected to ensure each individual was only recorded once. This experiment was performed at the beginning of July 2023 and was conducted three times.

### 2.6 Pollen analyses

In a further experiment, we determined the protein content of bee-collected pollen. For this purpose, pollen traps were installed in front of all ten colonies in June 2023. Pollen traps were custom-built from plywood, metal mesh, and a collection tray. The traps consisted of a wooden frame (approx. 44 x 10 × 10 cm) designed to fit a Zander hive system. A perforated metal entrance plate allows bees to pass while dislodging pollen, which was collected in a removable tray. Pollen was collected over a 24-h period and immediately frozen to preserve quality. Prior to analysis, the processed samples were freeze-dried and ground into a fine powder. The processed samples were then analyzed for their protein content using Bradford assays (Infinite 200 Pro, Tecan, Männedorf, Switzerland).

### 2.7 Honey harvest

Both spring and summer honey harvests were collected to determine the honey yield. Shortly before harvesting, honey bee escapes were placed between honey combs and brood combs to ensure that bees moved down into the brood area, leaving the honey frames gradually free of bees. Once the frames were cleared, they were removed from the colonies and weighed. The combined weight of boxes and built frames was subtracted to calculate the net honey yield. Honey yield was measured over a period of three years (for exact timing of honey harvests see Table S3).

### 2.8 Statistics

Statistical analyses were performed using R - v. 4.1.2 (R Core Team, 2021). Data handling and visualization were conducted with the packages “readxl - v. 1.3.1” (Wickham and Bryan, 2019), “tidyverse - v.1.3.1” (Wickham et al., 2019), “lubridate – v. 1.8.0”(Grolemund and Wickham, 2011), “dplyr - v. 1.0.7” (Wickham et al., 2021), “ggplot2 - v. 3.4.4” (Wickham, 2016) “ggpubr - v. 0.4.0” (Kassambara, 2020) and “ggeffects - v. 1.3.2” (Lüdecke, 2018). To analyze the effects of the beekeeping method on foraging behavior, juvenile hormone levels, pollen protein content, and honey yield, generalized linear mixed models (GLMMs) were applied using the glmmTMB package “glmmTMB - v. 1.1.7” (Brooks et al., 2017). The distribution family was selected individually for each model based on the best model fit. Model selection was guided by diagnostic checks, including residual analysis and goodness-of-fit measures. Residual diagnostics were performed using the DHARMa package “DHARMa - v. 0.4.5” (Hartig, 2022) to assess model assumptions such as normality and dispersion. Post hoc pairwise comparisons were conducted using “emmeans - v. 1.7.2” (Lenth, 2022), with Tukey's adjustment for multiple testing applied where appropriate. An overview of the applied statistical models, including the dependent variables, fixed and random effects, and distribution families, is provided in Table S1 in the supplementary material.

## 3 Results

### 3.1 Innovative beekeeping temporarily leads to high *Varroa* infestation

**Fig. 1:**
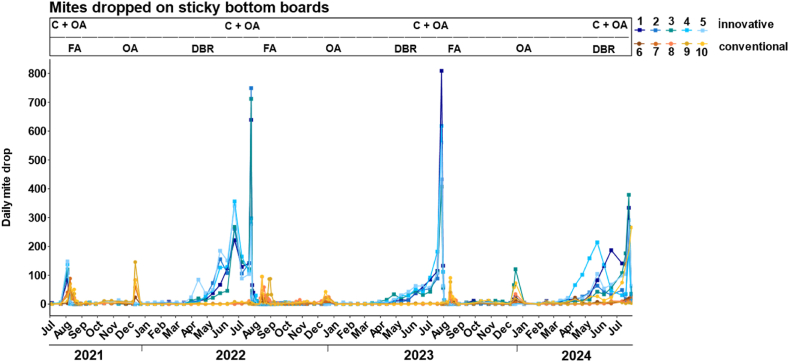
**Daily mite drop per colony from July 2021 to July 2024 in innovatively managed bee colonies (blue squares, colonies 1–5) and conventionally managed colonies (orange-yellow points, colonies 6–10).** The y-axis shows the number of dropped mites on sticky bottom boards per day, which were monitored at least every second week. The x-axis indicates the date in months and years. In July 2021, all colonies received their respective summer treatments for the first time (innovative: queen-caging (C) followed by oxalic acid treatment (OA); conventional: formic acid treatment (FA)). Omitting winter treatment and drone brood removal (DBR) led to a drastic increase in daily mite drop in spring and summer in innovatively managed bees (blue peaks). In December 2021, only the conventionally managed colonies were treated with oxalic acid (OA). The same treatment schedule was repeated in 2022 and 2023. In comparison to the previous two years, two colonies (colonies 1 and 3) of the innovative approach received an oxalic acid winter treatment in December 2023. In June 2024, the queens of the innovatively managed colonies were caged for the last time in this study, followed by an oxalic acid treatment in July 2024.

At the start of the experiment in July 2021, both groups had a moderate daily mite drop. In innovatively managed colonies, mite drop ranged from 84 to 148 dropped mites per day and was thus slightly higher than that in conventionally managed colonies, where daily mite drop ranged between 49 and 89 dropped mites (Fig. 1). At the beginning of the first winter (December 2021), only conventionally managed colonies received a winter treatment with oxalic acid, in line with the conventional beekeeping practice. This resulted in a distinct peak in daily mite drop, ranging from 24 to 146 mites per day. No winter treatment was applied to innovatively managed colonies in 2021.

The 2022 season represented the first full year under the respective beekeeping scheme. In the innovatively managed colonies, the lack of drone brood removal resulted in a drastic increase in daily mite drop in spring (Fig. 1). Following queen-caging in June 2022. The daily mite drop in innovatively managed colonies initially decreased slightly after caging. After oxalic acid treatment in July 2022, infestation values peaked sharply, with daily counts ranging from 278 to 749 dropped mites. In contrast, the formic acid treatment applied to conventionally managed colonies in August 2022 resulted in lower daily mite drop rates, ranging from 33 to 96. After the oxalic acid winter treatment in December 2022, the daily mite drop in conventionally managed colonies remained low, with daily counts between 8 and 43 dropped mites per day.

A similar trend was observed in 2023. At the beginning of the season, daily mite drop increased in innovatively managed colonies. After queen-caging and oxalic acid treatment in July, daily mite drop in innovatively managed colonies peaked again, with values ranging from 407 to 809 dropped mites daily. Summer treatment with formic acid in August 2023 resulted in daily mite drop rates between 35 and 91 in conventionally managed colonies. The oxalic acid winter treatment in December 2023 resulted in a daily mite drop between 7 and 73 mites per day in conventionally managed colonies. Additionally, two of the innovatively managed colonies (colonies 1 and 3) required oxalic acid treatment in winter 2023, resulting in a maximum of 25 dropped mites per day in colony 1 and a maximum of 121 mites per day in colony 3 after the treatment.

2024 again started with an increased daily mite drop in spring in the innovatively managed colonies. In July 2024, after the final queen-caging and oxalic acid treatment in the innovatively managed colonies, daily mite drop ranged from 182 to 379.

### 3.2 Foraging onset is advanced in innovatively compared to conventionally managed colonies

**Fig. 2:**
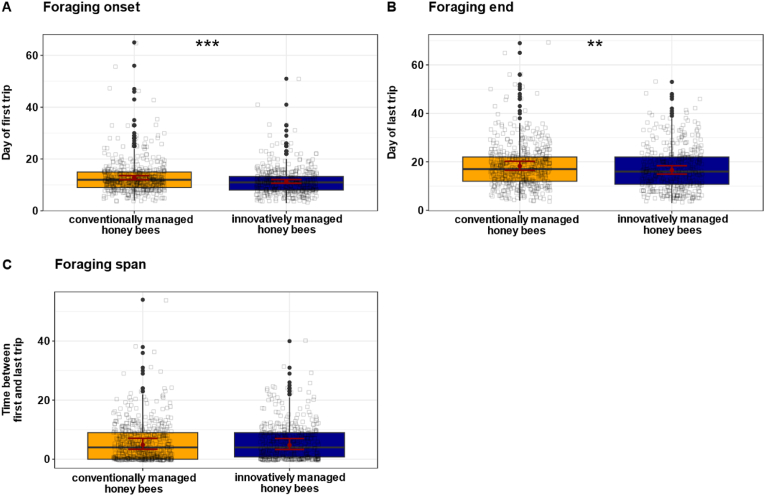
**Effect of conventional and innovative management on foraging onset (A), foraging end (B), and foraging span (C).** Boxplots show individual foraging onset, end, and span from honey bees from conventionally (n_conventional = 519, yellow) and innovatively managed colonies (n_innovative = 444, blue). Black squares represent individual honey bee data points. Red dots show the predicted mean values from the GLMM, and red bars indicate 95 % confidence intervals (CIs). *Varroa* pressure, which was elevated in the innovatively managed colonies, significantly influenced the foraging onset (χ^2^ = 19.6, p < 0.001), with bees from these colonies starting foraging earlier than those from conventionally managed colonies. The foraging end was also significantly influenced by *Varroa* pressure (χ^2^ = 10.6, p = 0.001), with bees from innovatively managed colonies stopping earlier compared to bees from conventionally managed colonies. *Varroa* pressure did not significantly affect the foraging span (χ^2^ = 0.062, p = 0.80). Significant differences between both groups are indicated by asterisks: ∗∗p < 0.01, ∗∗∗p < 0.001.

During experiments in June 2022 and 2023, when *Varroa* pressure was elevated in innovatively managed colonies, the onset of foraging was significantly affected (Fig. 2A; χ^2^ = 19.6, p < 0.001; n_innovative = 444, n_conventional = 519). Honey bees from innovatively managed colonies started foraging earlier (predicted mean ± confidence interval (CI): 11 days [10.7, 12.1]) compared to those from conventionally managed colonies (13 days [12.1, 13.6]). The end of foraging was also significantly influenced by *Varroa* pressure (Fig. 2B; χ^2^ = 10.6, p = 0.001; n_innovative = 444, n_conventional = 519). Honey bees from innovatively managed colonies stopped foraging earlier (17 days [15.0, 18.4]), whereas honey bees from conventionally managed colonies continued foraging until day 18 [16.5, 20.2]. Consequently, the foraging span did not differ significantly between the two groups (χ^2^ = 0.1, p = 0.80; n_innovative = 444, n_conventional = 519), with a predicted mean foraging span of 5 days [3.3, 7.0] in honey bees from innovatively managed colonies and 5 days [3.4, 7.1] in honey bees from conventionally managed colonies (Fig. 2C).

**Fig. 3:**
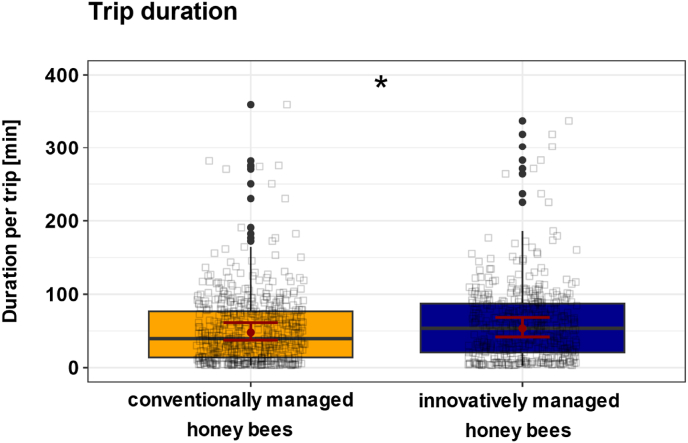
**Effect of conventional and innovative management on trip duration.** Boxplots show individual trip durations from honey bees from conventionally (n_conventional = 519, yellow) and innovatively managed colonies (n_innovative = 444, blue). Black squares represent individual honey bee data points. Red dots show the predicted mean values from the GLMM, and red bars indicate 95 % confidence intervals (CIs). *Varroa* pressure significantly influenced the trip duration (χ^2^ = 5.8, p = 0.02). Honey bees from innovatively managed colonies had longer trip durations compared to honey bees from conventionally managed colonies. The significant difference between the two groups is indicated by an asterisk: ∗p < 0.05.

Honey bees from innovatively managed colonies had longer trip durations in June 2022 and 2023, when *Varroa* pressure was elevated in these colonies, with a predicted mean trip duration of 54 min (95 % CI: 42, 68). In contrast, honey bees from conventionally managed colonies (95 % CI: 36, 61) had a predicted mean trip duration of 48 min (Fig. 3; χ^2^ = 5.8, p = 0.02; n_innovative = 444, n_conventional = 519).

**Fig. 4:**
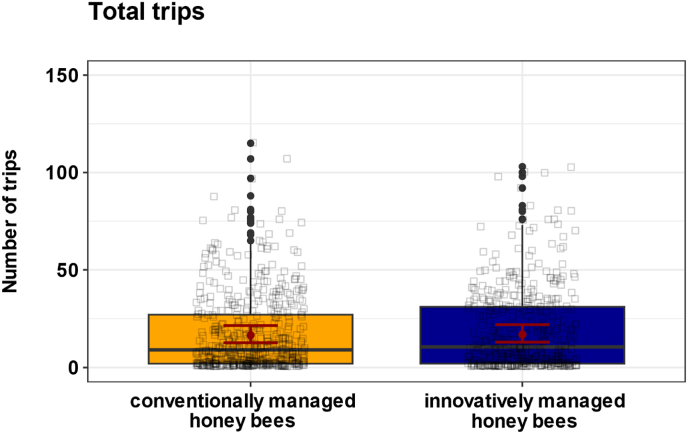
**Effect of conventional and innovative management on total trips.** Boxplots show individual trip durations from honey bees from conventionally (n = 519, yellow) and innovatively managed colonies (n = 444, blue). Black squares represent individual honey bee data points. Red dots show the predicted mean values from the GLMM, and red bars indicate 95 % confidence intervals (CIs). *Varroa* pressure had no significant effects on the total number of foraging trips (χ^2^ = 0.1, p = 0.71).

*Varroa* pressure, which was elevated in June 2022 and 2023 in innovatively managed colonies, had no significant effect on the total number of foraging trips performed by honey bees (Fig. 4; χ^2^ = 0.1, p = 0.71; n_innovative = 444, n_conventional = 519). Honey bees from innovatively managed colonies made an average of 16.8 trips (95 % CI: 13.0, 21.9) compared to 16.50 trips (95 % CI: 12.7, 21.4) from honey bees managed under conventional management.

### 3.3 Elevated *Varroa* pressure in innovatively managed colonies leads to a premature increase in juvenile hormone III

**Fig. 5:**
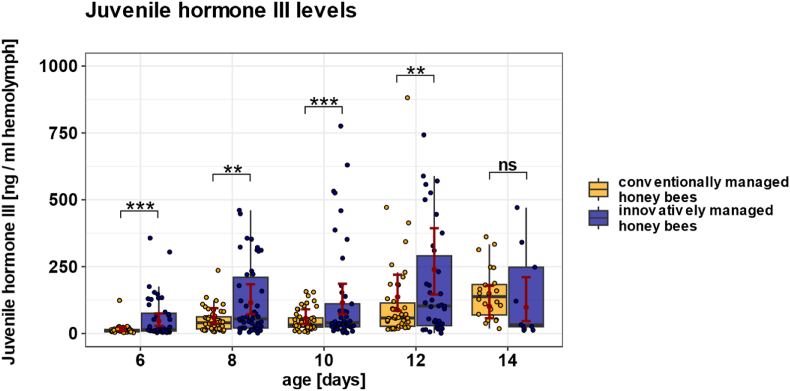
**Effect of conventional and innovative management on juvenile hormone III levels in the hemolymph.** Boxplots show individual juvenile hormone III levels from honey bees from conventionally (yellow) and innovatively managed colonies (blue). Filled colored dots represent individual honey bee data points. Red dots show the predicted mean values from the GLMM, and red bars indicate 95 % confidence intervals (CIs). Management and age both had significant effects on juvenile hormone III levels in the hemolymph. Juvenile hormone III levels differed significantly between both treatment groups (p < 0.001) and across ages (p < 0.001). Additionally, juvenile hormone III levels were significantly higher in honey bees from innovatively managed colonies at the age of 6 days (n_conventional = 40, n_innovative = 43, p < 0.001), 8 days (n_conventional = 48, n_innovative = 50, p = 0.002), 10 days (n_conventional = 49, n_innovative = 45, p = 0.001), and 12 days (n_conventional = 46, n_innovative = 38, p = 0.009). No significant difference was found between both groups at the age of 14 days (n_conventional = 24, n_innovative = 9, p = 0.95). Significant differences between both groups are indicated by asterisks: ∗p < 0.05; ∗∗p < 0.01, ∗∗∗p < 0.001.

*Varroa* pressure, elevated in innovatively managed colonies, and the age of honey bees both had significant effects on juvenile hormone III levels in the hemolymph. Juvenile hormone III titers differed significantly between the two treatment groups (χ^2^ = 42.0, p < 0.001; n_innovative = 185, n_conventional = 207) as well as across ages (Fig. 5; χ^2^ = 158.8, p < 0.001; 6 days, n_conventional = 40, n_innovative = 43; 8 days, n_conventional = 48, n_innovative = 50; 10 days, n_conventional = 49, n_innovative = 45; 12 days, n_conventional = 46, n_innovative = 38; 14 days, n_conventional = 24, n_innovative = 9). Post hoc comparisons revealed that juvenile hormone III levels were significantly higher in honey bees from innovatively managed colonies compared to those from the conventional management at 6 days of age (ratio = 0.32, p < 0.001), 8 days (ratio = 0.51, p = 0.002), 10 days (ratio = 0.49, p = 0.001), and 12 days (ratio = 0.57, p = 0.009). At 14 days of age, no significant difference was found between the two groups (ratio = 1.03, p = 0.95). However, at 14 days, very few marked honey bees from the group exposed to high *Varroa* pressure were found within the colonies, resulting in a low sample size (n = 9) for this age group.

### 3.4 Innovatively managed honey bees with elevated *Varroa* pressure do not show altered orientation

**Fig. 6:**
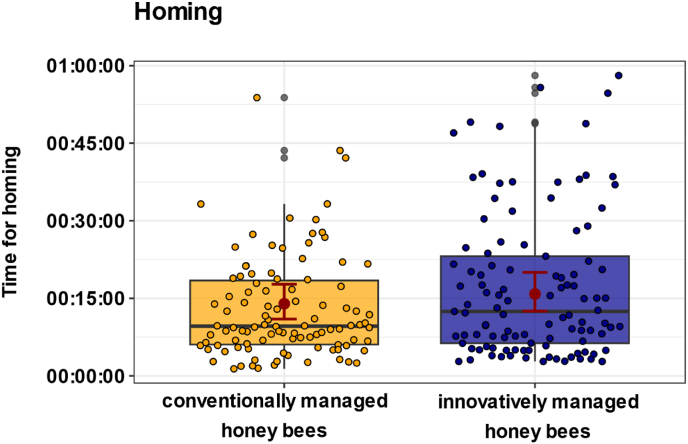
**Effect of conventional and innovative management on the time for homing.** Boxplots show individual homing times of honey bees from conventionally (n_conventional = 102, yellow) and innovatively managed colonies (n_innovative = 109, blue). Filled colored dots represent individual honey bee data points. Red dots show the predicted mean values from the GLMM, and red bars indicate 95 % confidence intervals (CIs). The elevated *Varroa* pressure of innovatively managed colonies had no significant effects on the homing time of honey bees (χ^2^ = 1.8, p = 0.18).

Innovative management did not affect the homing duration of foragers in early July 2023 (Fig. 6; χ^2^ = 1.8, p = 0.18; n_conventional = 102, n_innovative = 109). At this time, innovatively managed colonies experienced high mite infestation levels with an average daily mite drop of 66 mites per colony, whereas conventionally managed colonies showed only low infestations with an average daily mite drop of 2 mites per colony. Honey bees from innovatively managed colonies returned to the colony after an average of 16 min (95 % CI: 12.5, 20.0), whereas honey bees from conventionally managed colonies with lower *Varroa* pressure took an average of 14 min (95 % CI: 11.0, 17.7).

### 3.5 Innovatively managed colonies with elevated *Varroa* pressure collect more protein-rich pollen

**Fig. 7:**
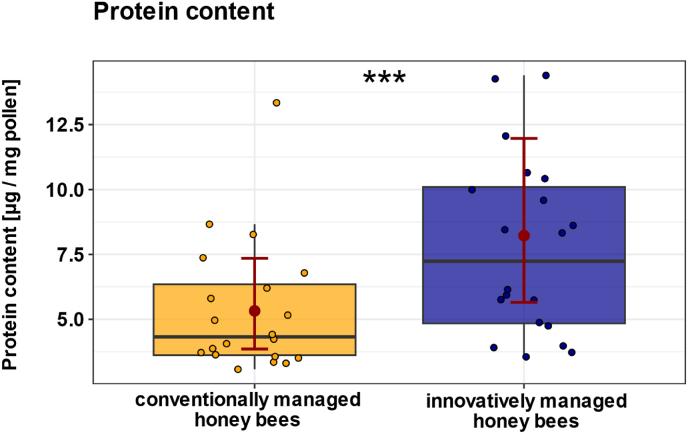
**Effect of conventional and innovative management on the protein content of collected pollen.** Boxplots show the protein content values of collected pollen from colonies under conventional (n_conventional = 20, yellow) and innovative management (n_innovative = 20, blue). Filled colored dots represent individual honey bee data points. Red dots show the predicted mean values from the GLMM, and red bars indicate 95 % confidence intervals (CIs). Management significantly affected the protein content of the collected pollen (p < 0.001). Honey bees from innovative management collected pollen with a higher protein content compared to honey bees from conventionally managed colonies. The significant difference between the two groups is indicated by an asterisk: ∗∗∗p < 0.001.

Because the homing abilities of innovatively managed honey bees were comparable to those of conventionally managed bees, we tested the hypothesis that longer foraging trip durations of these bees would be related to a search for pollen of high quality, i.e., pollen with a high protein content, for which bees might accept longer flight paths. Pollen was collected in mid-June 2023, when the average daily mite drop was 49 mites in innovatively managed colonies, compared to 2 mites in conventionally managed colonies. Innovative management accompanied by higher *Varroa* pressure had a significant effect on the protein content of pollen collected by foragers (Fig. 7; χ^2^ = 231.7, p < 0.001; n_conventional = 20, n_innovative = 20). Honey bees from innovatively managed colonies collected pollen with a higher protein content (predicted mean ± CI: 8.2 μg/mg pollen [5.7, 12.0]) compared to honey bees from conventionally managed colonies (predicted mean ± CI: 5.3 μg/mg pollen [3.9, 7.4]).

### 3.6 Honey yield does not differ between innovative and conventional beekeeping management

**Fig. 8:**
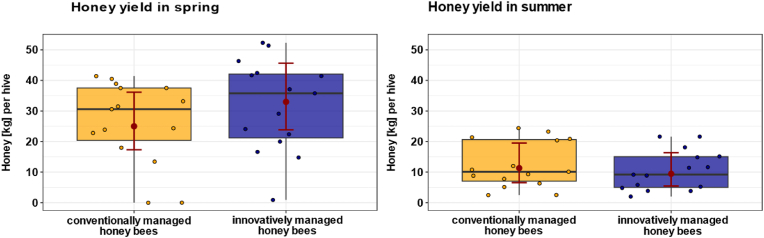
**Effect of *Varroa* pressure on honey yields in spring (A) and summer (B).** Boxplots show the honey yield in spring and summer from colonies under conventional (n_conventional = 15, yellow) and innovative management (n_innovative = 15, blue). Filled colored dots represent individual honey bee data points. Red dots show the predicted mean values from the GLMM, and red bars indicate 95 % confidence intervals (CIs). *Varroa* pressure had no significant effect on honey yield in spring and summer.

Honey yield was analyzed separately for the spring and summer harvests to evaluate the potential effects of conventional and innovative management on colony productivity across different seasons. In spring, honey yield did not differ between conventionally and innovatively managed colonies (Fig. 8; χ^2^ = 1.4, p = 0.24; n_conventional = 15, n_innovative = 15). Colonies under conventional management produced an average of 25 kg of honey (predicted mean ± CI: 25 kg of honey [17.3, 36.2]), whereas innovatively managed colonies produced an average of 33 kg (predicted mean ± CI: 33 kg of honey [23.9, 45.6]). At this time, *Varroa* pressure had started to increase in the innovatively managed colonies but remained low in the conventional colonies. Similarly, no significant difference was found in summer honey yield between the two groups (χ^2^ = 1.3, p = 0.25; n_conventional = 15, n_innovative = 15), although infestation was higher in innovatively managed colonies. Colonies under conventional management produced an average of 11 kg of honey (predicted mean ± CI: 11 kg of honey [6.6, 19.5]), whereas innovatively managed colonies produced an average of 9 kg of honey (predicted mean ± CI: 9 kg of honey [5.5, 16.4]).

## 4 Discussion

### 4.1 Reduced treatments against *Varroa* result in high mite loads in spring and summer

Reduced *Varroa* treatments in innovatively managed colonies by omitting drone brood removal during the reproductive season and refraining from winter oxalic acid treatments (unless a colony exceeded the *Varroa* threshold) led to temporarily higher *Varroa* pressure in spring and summer. This was particularly evident in these colonies, where daily mite drop peaked after summer treatment in 2022, 2023, and 2024, compared to the conventionally managed colonies, which received drone brood removal, formic acid summer treatment, and winter treatment with oxalic acid. Previous studies have shown that adaptation to *Varroa* can only occur when colonies are confronted with the parasite (Seeley, 2007; Locke, 2016). The innovative approach may represent a controlled exposure to *Varroa* pressure that fosters potential adaptive responses within the colonies. Traits such as *Varroa*-sensitive hygiene, recapping behavior, and reduced mite reproductive success have been identified in colonies surviving without human interventions and treatments against the mite (Panziera et al., 2017; Guichard et al., 2020; Grindrod and Martin, 2023). Although our colonies were not specifically selected for these traits, the innovative management strategy may offer a “middle ground” between conventional beekeeping, which involves numerous treatments, and natural selection approaches that use no treatments at all (Neumann and Blacquière, 2017; Brodschneider et al., 2022). Further research should focus on whether such controlled exposure can indeed promote adaptive traits in managed populations and whether thresholds for treatment intervention can be further optimized to balance colony health, vitality, and selective pressure.

### 4.2 *Varroa* pressure drives early foraging behavior and hormonal shifts

Honey bees from innovatively managed colonies initiated foraging earlier and also stopped foraging earlier than honey bees from conventionally managed colonies. The overall foraging span did not differ between the two groups. These shifts in foraging behavior were accompanied by significant changes in juvenile hormone titers in the hemolymph. Elevated juvenile hormone III levels were consistently found in younger honey bees (6–12 days old) from innovatively managed colonies. However, the comparison on day 14 is less reliable, because very few honey bees from this innovative group were still present in the colonies. The accelerated ontogeny of honey bees likely results from the interaction between *Varroa* parasitism and the endocrine system. *Varroa* feed on honey bee pupae during the reproduction phase, thereby significantly depleting their nutritional reserves (Bowen-Walker and Gunn, 2001; Nazzi and Le Conte, 2016; Han et al., 2024). Consequently, *Varroa*-infested honey bees emerge with reduced body mass and lower lipid stores (De Jong et al., 1982; Bowen-Walker and Gunn, 2001; Rosenkranz et al., 2010). In adult bees, *Varroa* preferentially target the fat body during the dispersal phases of their life cycle (Xie et al., 2016; Ramsey et al., 2019). The fat body in honey bees plays a crucial role, functioning as the main site of energy storage and as an endocrine and immune organ (Arrese and Soulages, 2010).

Based on previous studies, depletion of fat body tissue through parasitism may reduce vitellogenin production, which in turn could contribute to a shift in the endocrine balance toward increased juvenile hormone III titers (Amdam and Omholt, 2003; Page et al., 2012; Ramsey et al., 2019). Because vitellogenin and juvenile hormone III are inversely regulated, this mechanism may lead to an earlier and more pronounced increase in juvenile hormone III, potentially triggering precocious foraging, reduced immune function, and shortened lifespan, which aligns with previous studies (Robinson, 1987; Sullivan et al., 2000; Amdam and Omholt, 2003; Page et al., 2012; Hilsmann et al., 2025). These observations support the idea that the accelerated behavioral development we observed is accompanied by a shortened individual lifespan, because innovatively managed honey bees started foraging earlier and ended foraging sooner than honey bees from conventionally managed colonies. At 14 days of age, we could not find sufficient honey bees in the innovatively managed group for juvenile hormone analysis, further supporting the hypothesis of a reduced lifespan in this group. Early onset of foraging and shortened lifespan are well-established markers of colony stress responses (Perry et al., 2015).

While such patterns may represent a short-term compensatory mechanism aimed at maintaining colony productivity under stress conditions, they may also entail long-term costs for both the individual and colony resilience (Amdam and Page, 2005; Woyciechowski and Moroń, 2009; Perry et al., 2015). It remains unclear whether this response is purely stress-induced or whether it reflects early adaptive processes. For example, some *Varroa*-resistant populations exhibit shortened post-capping periods in brood development, which limits mite reproductive success (Harbo, 1992; Oddie et al., 2018; Guichard et al., 2020). It is conceivable that the accelerated maturation observed in adult honey bees may be linked to similar processes at the brood level. Whereas our colonies were not selected for resistance traits such as *Varroa*-sensitive hygiene or shortened post-capping periods, acceleration of behavioral maturation raises the question of whether such traits could emerge under high mite pressure. However, further research is needed to clarify whether such traits are expressed under reduced-treatment strategies and how they might contribute to colony-level resistance. It is worth noting that despite the early onset of foraging, radio frequency identification-tagged honey bees were transferred to Mini-Plus colonies, which might have moderated the expression of behavioral and colony-level stress responses. Nevertheless, acceleration in foraging onset and physiological maturation appears to reflect a realistic consequence of elevated *Varroa* pressure.

### 4.3 Prolonged foraging duration for better pollen sources

In our study, honey bees from the innovative beekeeping approach, exposed to higher *Varroa* infestation, took longer foraging trip durations. Previous studies have suggested that *Varroa* infestation impairs orientation and navigation ability (Kralj and Fuchs, 2006). However, our homing experiments did not reveal significant differences between the two treatment groups. Instead, our pollen analyses suggest an alternative explanation. Bees from innovatively managed colonies collected pollen with significantly higher protein content. Ghosh et al. (2020) showed that honey bees can discriminate between pollen sources. Therefore, these honey bees might have extended their foraging trips to locate higher-quality floral resources as a compensatory strategy to mitigate the physiological damage caused by *Varroa*. In general, pollen can serve as an essential resource to compensate for damages caused by *Varroa* infestation, and adequate pollen availability is known to be crucial for maintaining colony performance and survival (Annoscia et al., 2017; Requier et al., 2017). In line with this, high-protein pollen diets are known to counteract the negative effects of *Varroa* on immune function and longevity (Alaux et al., 2010; Huang, 2012; Di Pasquale et al., 2013; Piou et al., 2018; Frizzera et al., 2022). The increased protein content in pollen collected by these honey bees could therefore reflect an adaptive foraging strategy to improve nutritional intake under stressful conditions, such as *Varroa* infestation and accompanied viral infections (Hilsmann et al., 2025). Notably, despite the longer trip durations and earlier foraging onset, the total number of foraging trips was not significantly different between the two groups. Furthermore, there was no significant difference in honey yield between colonies managed conventionally or innovatively, either in spring or summer. This is in line with a previous study by Kovačić et al. (2023), who showed that despite an induced brood interruption, honey production was not negatively impacted, provided the caging was not performed too early before the honey harvest. Importantly, in our study, summer honey harvest was completed before caging the queen (for detailed dates see Table S2), and honey yield reported only includes frames from the honey super, ensuring that caging and brood manipulation did not influence the measured production. This suggests that colonies exposed to higher mite loads during the active season can maintain their foraging activity and productivity, possibly due to compensatory behavioral adaptations.

## 5 Conclusion

Our study shows that honey bee colonies managed with an innovative approach, leading to temporarily elevated *Varroa* pressure, exhibited significant changes in foraging behavior and physiology. Workers from these colonies initiated foraging earlier and showed elevated juvenile hormone levels at younger ages. Their foraging trips were longer, although their orientation abilities were not different between honey bees from both beekeeping approaches. But foragers from these colonies collected pollen with higher protein content, suggesting a potential shift in resource selection under elevated mite pressure. Colony productivity remained unaffected despite these behavioral and physiological shifts, including an earlier end of foraging activity that may indicate a reduced individual lifespan. These findings suggest that honey bee colonies may compensate for the temporary stress of high *Varroa* pressure through behavioral and physiological adjustments. However, whether these responses represent sustainable long-term strategies remains unclear. Further studies are needed to investigate whether temporary exposure to elevated *Varroa* pressure can influence the expression of established resistance traits such as *Varroa*-sensitive hygiene or recapping behavior. Understanding whether and how these traits develop under reduced-treatment conditions could provide valuable insights into potential adaptive processes in managed colonies. Additionally, the finding that foragers from innovatively managed colonies under higher mite pressure collected pollen with a higher protein content raises new questions about their foraging decisions. Identifying the plant species or floral sources involved could help clarify whether bees actively select nutritionally richer pollen as a compensatory mechanism to reduce effects of *Varroa*. Altogether, our results highlight the potential of the innovative beekeeping approach to balancing effective *Varroa* management with colony vitality while opening up new avenues for research into compensatory foraging behavior and the potential development of *Varroa* resistance traits.

## CRediT authorship contribution statement

**Lioba Hilsmann:** Writing – review & editing, Writing – original draft, Visualization, Investigation, Formal analysis, Data curation, Conceptualization. **Markus Krischke:** Writing – review & editing, Methodology, Investigation. **Martin J. Mueller:** Writing – review & editing, Supervision, Resources. **Sarah Manzer:** Writing – review & editing, Visualization, Formal analysis. **Ricarda Scheiner:** Writing – review & editing, Writing – original draft, Supervision, Resources, Project administration, Funding acquisition, Conceptualization.

## Ethics statement

Our protocols comply with standard welfare practices in our field.

## Publisher's note

All claims expressed in this article are solely those of the authors and do not necessarily represent those of their affiliated organizations or those of the publisher, the editors, and the reviewers. Any product that may be evaluated in this article or claim that may be made by its manufacturer is not guaranteed or endorsed by the publisher.

## Declaration of generative AI and AI-assisted technologies in the writing process

During the preparation of this work the author(s) used ChatGPT (OpenAI) and Grammarly (Grammarly Inc.) in order to improve the language. After using this tool/service, the author(s) reviewed and edited the content as needed and take(s) full responsibility for the content of the publication.

## Funding

This work was supported by funds from the 10.13039/501100005908Federal Ministry of Food and Agriculture (BMEL) based on a decision of the parliament of the Federal Republic of Germany via the 10.13039/501100010473Federal Office for Agriculture and Food (BLE): 2819NA036.

## Declaration of competing interest

The authors declare that the research was conducted without any commercial or financial relationship that could be construed as a potential conflict of interest.
