# Supplementary material for: Balancing Varroa management and honey bee resilience: Behavioral and physiological consequences of temporarily high mite pressure
Source: Int J Parasitol Parasites Wildl. 2025 Sep 13;28:101137. doi: 10.1016/j.ijppaw.2025.101137 (PMC12481707; doi:10.1016/j.ijppaw.2025.101137)
Supplement: Multimedia component 1 [file mmc1.docx]

**Table S1: Summary of models used in the analysis of data.** The dependent variable, model, and family are provided for each model. The family was selected based on the best-fitting model.

| Dependent Variable | Model | Family |
| --- | --- | --- |
| Foraging onset | glmmTMB(onset ~ treatment+(1\|replicat/year) | nbinom1 |
| Foraging end | glmmTMB(end ~ treatment+(1\|replicat/year) | nbinom1 |
| Foraging span | glmmTMB(span ~ treatment+(1\|replicat/year) | nbinom1 |
| Trip duration | glmmTMB(duration_per_trip_min~treatment+(1\|replicat/year) | nbinom1 |
| Total trips | glmmTMB(total_trips ~ treatment+(1\|replicat/year) | nbinom1 |
| Juvenile hormone | glmmTMB(JH ~ treatment*age+(1\|rep/year) | nbinom2 |
| Homing | glmmTMB(time_sec ~ treatment+(1\|rep/hive)t | nbinom1 |
| Pollen proteins | glmmTMB(proteins ~ treatment+(1\|rep/hive) | nbinom1 |
| Honey yield | glmmTMB(honey ~ treatment+(1\|year)+(1\|hive) | nbinom1 |

**Table S2: Treatment scheme.** The table summarizes the exact dates and details of all *Varroa* treatments.

| Year | 1^st^ Drone brood removal | Caging of the queen | Oxalic acid treatment summer | Formic acid treatment summer | Oxalic acid treatment winter |
| --- | --- | --- | --- | --- | --- |
| 2021 | --- | 08.07.2021 | 02.08.2021 | 02.08.2021 | 10.12.2021 |
| 2022 | 18.05.2022 | 28.06.2022 | 22.07.2022 | 12.08.2022 | 13.12.2022 |
| 2023 | 04.05.2023 | 30.06.2023 | 25.07.2023 | 10.08.2023 | 13.12.2023 |
| 2024 | 24.04.2024 | 28.06.2024 | 22.07.2024 | --- | --- |

**Table S3: Dates of honey harvests.** The table summarizes the exact dates of honey harvests.

| Year | Spring | Summer |
| --- | --- | --- |
| 2022 | 19.05.2022  31.05.2022 | 08.07.2022 |
| 2023 | 14.06.2023 | 20.07.2023 |
| 2024 | 15.05.2024 | 12.07.2024 |
